# Supplementary material for: Resolving the full spectrum of human genome variation using Linked-Reads
Source: Genome Res. 2019 Apr;29(4):635–45. doi: 10.1101/gr.234443.118 (PMC6442396; doi:10.1101/gr.234443.118)
Supplement: Supplemental Material [file supp_gr.234443.118_Supplemental_longranger-2.2.zip › longranger-2.2.tar.bz2!/lr2.2-public/lib/stan/cmdstan/stan/doc/api/html/structstan_1_1gm_1_1bare__type__grammar.html]

Stan: stan::gm::bare\_type\_grammar< Iterator > Struct Template Reference


|  |  |
| --- | --- |
|  | Stan  2.5.0  probability, sampling & optimization |


- Main Page
- Related Pages
- Namespaces
- Classes
- Files

- Class List
- Class Index
- Class Hierarchy
- Class Members

All Classes Namespaces Files Functions Variables Typedefs Enumerations Enumerator Friends Macros Pages

- stan
- gm
- bare\_type\_grammar

Public Member Functions |
Public Attributes |
List of all members

stan::gm::bare\_type\_grammar< Iterator > Struct Template Reference

`#include <bare_type_grammar.hpp>`

Inheritance diagram for stan::gm::bare\_type\_grammar< Iterator >:

|  |  |
| --- | --- |
| Public Member Functions | |
|  | bare\_type\_grammar (variable\_map &var\_map, std::stringstream &error\_msgs) |
|  | |

|  |  |
| --- | --- |
| Public Attributes | |
| variable\_map & | var\_map\_ |
|  | |
| std::stringstream & | error\_msgs\_ |
|  | |
| boost::spirit::qi::rule  < Iterator, expr\_type(),   whitespace\_grammar< Iterator > > | bare\_type\_r |
|  | |
| boost::spirit::qi::rule  < Iterator, base\_expr\_type(),   whitespace\_grammar< Iterator > > | type\_identifier\_r |
|  | |
| boost::spirit::qi::rule  < Iterator, size\_t(),   whitespace\_grammar< Iterator > > | array\_dims\_r |
|  | |

## Detailed Description

### template<typename Iterator> struct stan::gm::bare\_type\_grammar< Iterator >

Definition at line 19 of file bare\_type\_grammar.hpp.

## Constructor & Destructor Documentation

template<typename Iterator >

|  |  |  |  |
| --- | --- | --- | --- |
| stan::gm::bare\_type\_grammar< Iterator >::bare\_type\_grammar | ( | variable\_map & | *var\_map*, |
|  |  | std::stringstream & | *error\_msgs* |
|  | ) |  |  |

Definition at line 37 of file bare\_type\_grammar\_def.hpp.

## Member Data Documentation

template<typename Iterator >

boost::spirit::qi::rule<Iterator, size\_t(), whitespace\_grammar<Iterator> > stan::gm::bare\_type\_grammar< Iterator >::array\_dims\_r

Definition at line 45 of file bare\_type\_grammar.hpp.

template<typename Iterator >

boost::spirit::qi::rule<Iterator, expr\_type(), whitespace\_grammar<Iterator> > stan::gm::bare\_type\_grammar< Iterator >::bare\_type\_r

Definition at line 35 of file bare\_type\_grammar.hpp.

template<typename Iterator >

std::stringstream& stan::gm::bare\_type\_grammar< Iterator >::error\_msgs\_

Definition at line 26 of file bare\_type\_grammar.hpp.

template<typename Iterator >

boost::spirit::qi::rule<Iterator, base\_expr\_type(), whitespace\_grammar<Iterator> > stan::gm::bare\_type\_grammar< Iterator >::type\_identifier\_r

Definition at line 40 of file bare\_type\_grammar.hpp.

template<typename Iterator >

variable\_map& stan::gm::bare\_type\_grammar< Iterator >::var\_map\_

Definition at line 25 of file bare\_type\_grammar.hpp.

---

The documentation for this struct was generated from the following files:

- src/stan/gm/grammars/bare\_type\_grammar.hpp
- src/stan/gm/grammars/bare\_type\_grammar\_def.hpp


---

[ Stan Home Page ]

*© 2011–2014,
Stan Development Team.*
